# Supplementary material for: Modeling glioblastoma heterogeneity as a dynamic network of cell states
Source: Mol Syst Biol. 2021 Sep 16;17(9):e10105. doi: 10.15252/msb.202010105 (PMC8444284; doi:10.15252/msb.202010105)
Supplement: Supplementary file 6 — Source Data for Figure 5 [file MSB-17-e10105-s004.zip › Figure5A_sourcedata/GSEA_3071/nosigdb_stateVI.GseaPreranked.1622795169056/RICHARDS_NOWAKOWSKI_SCIENCE_2017_NIN1_UPREG.html]

Details for gene set RICHARDS\_NOWAKOWSKI\_SCIENCE\_2017\_NIN1\_UPREG[GSEA]

|  || Dataset | state63071 |
| Phenotype | NoPhenotypeAvailable |
| Upregulated in class | na\_neg |
| GeneSet | RICHARDS\_NOWAKOWSKI\_SCIENCE\_2017\_NIN1\_UPREG |
| Enrichment Score (ES) | -0.59380054 |
| Normalized Enrichment Score (NES) | -2.8216107 |
| Nominal p-value | 0.0 |
| FDR q-value | 0.0 |
| FWER p-Value | 0.0 |
Table: GSEA Results Summary

  

Fig 1: Enrichment plot: RICHARDS\_NOWAKOWSKI\_SCIENCE\_2017\_NIN1\_UPREG      
 Profile of the Running ES Score & Positions of GeneSet Members on the Rank Ordered List

  

| PROBE | GENE SYMBOL | GENE\_TITLE | RANK IN GENE LIST | RANK METRIC SCORE | RUNNING ES | CORE ENRICHMENT || 1 | RND3 |  |  | 49 | 0.866 | 0.0008 | No |
| 2 | DIMT1 |  |  | 273 | 0.552 | -0.0526 | No |
| 3 | ADAMTS5 |  |  | 487 | 0.458 | -0.1048 | No |
| 4 | ACTG1 |  |  | 508 | 0.453 | -0.1028 | No |
| 5 | ZNF618 |  |  | 709 | 0.395 | -0.1524 | No |
| 6 | PDE4DIP |  |  | 1408 | 0.291 | -0.3437 | No |
| 7 | ZC3H8 |  |  | 1439 | 0.286 | -0.3473 | No |
| 8 | EIF1AX |  |  | 1657 | 0.263 | -0.4039 | No |
| 9 | ATF2 |  |  | 1816 | -0.251 | -0.4441 | No |
| 10 | KIAA1958 |  |  | 1878 | -0.261 | -0.4568 | No |
| 11 | MOB3B |  |  | 1883 | -0.261 | -0.4536 | No |
| 12 | RC3H2 |  |  | 1930 | -0.269 | -0.4620 | No |
| 13 | PCDH17 |  |  | 1955 | -0.271 | -0.4642 | No |
| 14 | GNPTAB |  |  | 1971 | -0.274 | -0.4638 | No |
| 15 | CBX3 |  |  | 1983 | -0.275 | -0.4622 | No |
| 16 | MTRNR2L1 |  |  | 2113 | -0.300 | -0.4935 | No |
| 17 | FAM107B |  |  | 2234 | -0.320 | -0.5218 | No |
| 18 | MIR181A1HG |  |  | 2238 | -0.321 | -0.5173 | No |
| 19 | ARID4B |  |  | 2282 | -0.330 | -0.5238 | No |
| 20 | TIAM1 |  |  | 2303 | -0.334 | -0.5238 | No |
| 21 | STAT2 |  |  | 2345 | -0.342 | -0.5296 | No |
| 22 | DMTF1 |  |  | 2445 | -0.366 | -0.5513 | No |
| 23 | GLCCI1 |  |  | 2521 | -0.383 | -0.5659 | No |
| 24 | SENP7 |  |  | 2586 | -0.396 | -0.5772 | No |
| 25 | DST |  |  | 2614 | -0.404 | -0.5780 | No |
| 26 | MAF |  |  | 2633 | -0.408 | -0.5762 | No |
| 27 | LINC00342 |  |  | 2657 | -0.413 | -0.5757 | No |
| 28 | DCAF7 |  |  | 2663 | -0.414 | -0.5702 | No |
| 29 | PDZRN3 |  |  | 2685 | -0.421 | -0.5690 | No |
| 30 | ST8SIA4 |  |  | 2719 | -0.430 | -0.5710 | No |
| 31 | ELF2 |  |  | 2733 | -0.434 | -0.5674 | No |
| 32 | ELAVL3 |  |  | 2822 | -0.463 | -0.5843 | No |
| 33 | QKI |  |  | 2844 | -0.474 | -0.5823 | No |
| 34 | PDZRN4 |  |  | 2886 | -0.490 | -0.5856 | Yes |
| 35 | ANTXR1 |  |  | 2893 | -0.492 | -0.5790 | Yes |
| 36 | BEST3 |  |  | 2911 | -0.498 | -0.5753 | Yes |
| 37 | PARP1 |  |  | 2918 | -0.501 | -0.5686 | Yes |
| 38 | ARX |  |  | 2960 | -0.520 | -0.5714 | Yes |
| 39 | KLHL13 |  |  | 3000 | -0.542 | -0.5732 | Yes |
| 40 | TUBB3 |  |  | 3044 | -0.560 | -0.5759 | Yes |
| 41 | SOX4 |  |  | 3066 | -0.571 | -0.5722 | Yes |
| 42 | ARL4D |  |  | 3076 | -0.577 | -0.5650 | Yes |
| 43 | B4GALT5 |  |  | 3115 | -0.595 | -0.5657 | Yes |
| 44 | NRXN3 |  |  | 3127 | -0.603 | -0.5586 | Yes |
| 45 | MEIS1 |  |  | 3133 | -0.610 | -0.5498 | Yes |
| 46 | CADPS |  |  | 3161 | -0.629 | -0.5468 | Yes |
| 47 | PFN2 |  |  | 3215 | -0.685 | -0.5502 | Yes |
| 48 | CDCA7 |  |  | 3225 | -0.693 | -0.5410 | Yes |
| 49 | CDK6 |  |  | 3234 | -0.704 | -0.5314 | Yes |
| 50 | BCL11A |  |  | 3236 | -0.705 | -0.5199 | Yes |
| 51 | FNBP1L |  |  | 3237 | -0.708 | -0.5080 | Yes |
| 52 | RBP1 |  |  | 3264 | -0.723 | -0.5031 | Yes |
| 53 | GAD1 |  |  | 3266 | -0.726 | -0.4912 | Yes |
| 54 | BCL11B |  |  | 3276 | -0.735 | -0.4813 | Yes |
| 55 | DLX6 |  |  | 3289 | -0.749 | -0.4721 | Yes |
| 56 | CCNE2 |  |  | 3305 | -0.767 | -0.4634 | Yes |
| 57 | SOX2 |  |  | 3314 | -0.778 | -0.4526 | Yes |
| 58 | SRGAP2B |  |  | 3372 | -0.841 | -0.4544 | Yes |
| 59 | SLAIN1 |  |  | 3385 | -0.868 | -0.4432 | Yes |
| 60 | ZEB2 |  |  | 3395 | -0.881 | -0.4309 | Yes |
| 61 | MAPK10 |  |  | 3398 | -0.886 | -0.4166 | Yes |
| 62 | SP9 |  |  | 3411 | -0.914 | -0.4046 | Yes |
| 63 | CCDC88A |  |  | 3421 | -0.944 | -0.3912 | Yes |
| 64 | GOLM1 |  |  | 3435 | -0.972 | -0.3785 | Yes |
| 65 | SOX11 |  |  | 3443 | -0.989 | -0.3638 | Yes |
| 66 | ZNF704 |  |  | 3448 | -1.004 | -0.3481 | Yes |
| 67 | CHD7 |  |  | 3454 | -1.017 | -0.3324 | Yes |
| 68 | NEDD4L |  |  | 3469 | -1.045 | -0.3187 | Yes |
| 69 | RAB3IP |  |  | 3493 | -1.094 | -0.3068 | Yes |
| 70 | NCAM1 |  |  | 3514 | -1.168 | -0.2927 | Yes |
| 71 | SOX6 |  |  | 3517 | -1.174 | -0.2735 | Yes |
| 72 | DPYSL3 |  |  | 3521 | -1.188 | -0.2544 | Yes |
| 73 | DCLK2 |  |  | 3524 | -1.206 | -0.2347 | Yes |
| 74 | TCF4 |  |  | 3528 | -1.214 | -0.2151 | Yes |
| 75 | LSAMP |  |  | 3537 | -1.257 | -0.1962 | Yes |
| 76 | CDH2 |  |  | 3543 | -1.297 | -0.1758 | Yes |
| 77 | ZBTB20 |  |  | 3567 | -1.435 | -0.1581 | Yes |
| 78 | DLX1 |  |  | 3593 | -1.696 | -0.1366 | Yes |
| 79 | DLX2 |  |  | 3595 | -1.720 | -0.1079 | Yes |
| 80 | PLXNA4 |  |  | 3598 | -1.782 | -0.0785 | Yes |
| 81 | DLX5 |  |  | 3623 | -2.501 | -0.0431 | Yes |
| 82 | DCX |  |  | 3630 | -2.797 | 0.0022 | Yes |
Table: GSEA details [plain text format]

  

Fig 2: RICHARDS\_NOWAKOWSKI\_SCIENCE\_2017\_NIN1\_UPREG: Random ES distribution      
 Gene set null distribution of ES for **RICHARDS\_NOWAKOWSKI\_SCIENCE\_2017\_NIN1\_UPREG**
